# The Implementation of TNFRSF Co-Stimulatory Domains in CAR-T Cells for Optimal Functional Activity

**DOI:** 10.3390/cancers14020299

**Published:** 2022-01-08

**Authors:** Yuan He, Martijn Vlaming, Tom van Meerten, Edwin Bremer

**Affiliations:** 1 Department of Basic Medicine, School of Basic Medicine and Clinical Pharmacy, China Pharmaceutical University, Nanjing 211198, China; yuanhe0822@cpu.edu.cn; 2 Department of Hematology, University Medical Center Groningen, University of Groningen, 9713 GZ Groningen, The Netherlands; m.r.vlaming@umcg.nl (M.V.); t.van.meerten@umcg.nl (T.v.M.)

**Keywords:** 4-1BB, OX40, CD40, CD27, GITR, HVEM, CAR-T cell

## Abstract

**Simple Summary:**

Members of the Tumor Necrosis Factor Receptor Superfamily (TNFRSF) provide crucial co-stimulatory signals to many if not all immune effector cells. With distinct and unique functional features on multiple types of immune effector cells, the co-stimulatory activity of TNFRSF members is being implemented in the tailoring of Chimeric Antigen Receptor (CAR) T cell activity for cancer therapy. This integration of intracellular TNFRSF stimulatory domains into a CAR provides a unique signaling output. Here, we highlight the rationale and summarize the current evidence for the application and the unique attributes of co-stimulatory signaling by TNFRSF members (4-1BB; OX40; CD40; CD27; GITR; HVEM) in CAR-T therapy.

**Abstract:**

The Tumor Necrosis Factor Receptor Superfamily (TNFRSF) is a large and important immunoregulatory family that provides crucial co-stimulatory signals to many if not all immune effector cells. Each co-stimulatory TNFRSF member has a distinct expression profile and a unique functional impact on various types of cells and at different stages of the immune response. Correspondingly, exploiting TNFRSF-mediated signaling for cancer immunotherapy has been a major field of interest, with various therapeutic TNFRSF-exploiting anti-cancer approaches such as 4-1BB and CD27 agonistic antibodies being evaluated (pre)clinically. A further application of TNFRSF signaling is the incorporation of the intracellular co-stimulatory domain of a TNFRSF into so-called Chimeric Antigen Receptor (CAR) constructs for CAR-T cell therapy, the most prominent example of which is the 4-1BB co-stimulatory domain included in the clinically approved product Kymriah. In fact, CAR-T cell function can be clearly influenced by the unique co-stimulatory features of members of the TNFRSF. Here, we review a select group of TNFRSF members (4-1BB, OX40, CD27, CD40, HVEM, and GITR) that have gained prominence as co-stimulatory domains in CAR-T cell therapy and illustrate the unique features that each confers to CAR-T cells.

## 1. Introduction

Chimeric Antigen Receptor (CAR)-T cell therapy has emerged as perhaps the most significant breakthrough in immunotherapy in the past decade, particularly based on high complete remission rates in B cell malignancies in previously end-stage patients [1,2,3]. The road to this successful implementation of CAR-T in clinical practice has been long and only made possible by innovations in the composition of the CAR construct, particularly of the intracellular domain. In brief, first generation (G1) CAR-T cell constructs contained a high-affinity single-chain variable fragment (scFv) for the tumor-associated antigen, a transmembrane domain, and a sole intracellular TCR-ζ or FcR-γ domain [4,5]. G1 CAR-T cells had limited clinical benefit, mainly due to their low proliferative capacity and limited in vivo cytotoxicity [6,7]. To overcome this drawback, so-called second generation (G2) CAR constructs were equipped with an additional intracellular co-stimulatory domain, most notably CD28 or the Tumor Necrosis Factor Receptor Superfamily (TNFRSF) member 4-1BB (or CD137). These G2 CARs have proven to be very effective, with Yescarta (a CD28 G2), Kymriah (a 4-1BB G2), BREYANZI (a 4-1BB G2), TECARTUS (a CD28 G2), and ABECMA (a 4-1BB G2) receiving regulatory approval for the treatment of several hematological malignancies.

**Figure 1:**
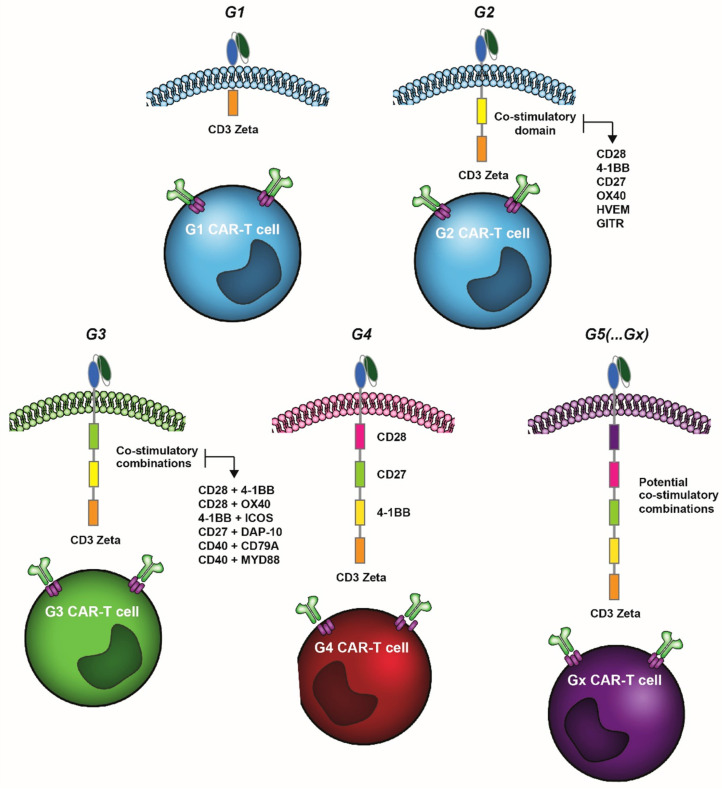
Graphic illustration of different generations of CAR-T cell therapy containing none, one, or a multitude of distinct co-stimulatory TNFRSF members. Note: as referred to in the text G4 and Gx CAR-T may contain other adaptor molecules, such as pro-inflammatory cytokines and switch receptors, not depicted here.

To date, the majority of clinical trials is still performed with G2 CAR-T cells. Nevertheless, these G2 versions often do not trigger complete responses in many cancers, with even success stories such as DLBCL having a ~50% relapse rate upon CD19 G2 CAR-T treatment. Therefore, efforts have focused on improving the activity, specificity, and in vivo control of CAR-T cells. This includes, on the one hand, strategies to optimize tumor targeting with the use of dual CARs, triple CARs, split CARs, inducible-split CARs, switchable CARs, and universal CARs (as reviewed in [8,9,10]). On the other hand, a major focus has been the further engineering of the intracellular stimulatory CAR domain, with a recent report describing a combinatorial library of 700k intracellular domains to screen for optimal ICD composition [11]. In this respect, third generation (G3) CAR-T constructs containing both the CD28 and 4-1BB co-stimulatory domains were reported [12,13,14], whereas fourth (G4) and fifth (G5) generation CAR-T cells contain either additional co-stimulatory domains, inducibly express chemokines upon antigen recognition (e.g., pro-inflammatory IL-12), or contain the intracellular domain of certain cytokine receptors (e.g., a truncated IL-2 receptor β chain and a STAT3-binding moiety) [8] (Figure 1).

As evident from the data gathered with G2 CD19 CARs, the use of a 4-1BB or CD28 intracellular domain confers distinct functional attributes to CAR-T cells, with the in vivo persistence of CAR-T cells being augmented by the 4-1BB domain [15,16,17,18]. Conversely, the CD28 domain more robustly activates the initial effector functions of CAR-T cells [15,16,19]. Thus, rational choice and further engineering of the intracellular CAR domain provides a clear and as yet incompletely explored means of fine-tuning and tailoring CAR-T cell activities, with various TNFRSF domains that have already been preclinically evaluated in CAR-T design [10,20]. In fact, other TNFRSF co-stimulatory domains, such as OX40, CD27, CD40, HVEM, and GITR, may be superior CAR-T cell drivers [10]. 

Here, we provide a review of the use of the intracellular co-stimulatory domains of the TNFR superfamily to augment and fine-tune CAR-T cell activity. Each of these TNFRSF superfamily members has overlapping as well as distinctive features that can be rationally applied to tailor therapeutic activity. 

## 2. 4-1BB as Co-Stimulatory Domain in CAR-T Cells

4-1BB is a key co-stimulatory receptor that is rapidly upregulated on T cells upon TCR–MHC interaction [21,22]. Its ligand 4-1BBL is expressed on antigen-presenting cells such as macrophages and dendritic cells [23,24]. The ligation of 4-1BB by 4-1BBL leads to the recruitment of TRAF1, TRAF2, and TRAF3 to the intracellular cytoplasmic domain of 4-1BB. Subsequently, a 4-1BB signaling complex or signalosome comprising a multitude of proteins, including kinases and ubiquitin ligases, is formed and ultimately initiates key signaling pathways such as NF-kB and PI3K-AKT (reviewed in [25]). Eventually, 4-1BB signaling induces the proliferation of cytotoxic T cells, the secretion of pro-inflammatory cytokines, and the expansion of effector and memory T cells (Tm) [26] (for an in-depth review, see [27]). 

The cytoplasmic signaling domain of 4-1BB is the earliest applied and most explored of the TNFRSF members to be included in CAR-T cell constructs [28,29,30,31,32]. Compared to CD28 CAR-T cells, 4-1BB co-stimulation yields CAR-T cells with distinct metabolic characteristics such as the reduced expression of GLUT1 on the T cell surface and increased mitochondrial respiration [15,33]. These differences are also reflected in the CAR-T cell phenotype and functionality. For instance, 4-1BB co-stimulation in CD19-targeting CAR-T cells yielded a more potent clearance of B-ALL in a mouse model, and 4-1BB CAR-T persisted longer than CD28 co-stimulated CAR T cells [34]. 

Moreover, 4-1BB co-stimulation favorably promoted the expansion of CD8 central memory CAR-T cells and provided a better in vivo survival and proliferation profile [15,16,17,18]. Notably, 4-1BB CAR-T cell preparations also contained higher levels of stem cell memory T cells than CD28 CAR-T cells [35,36,37]. This CAR-T differentiation status has been linked to the better in vivo expansion of CAR-T cells and is considered a predictive indicator of clinical response [35,36,37]. Furthermore, co-stimulatory domains also impact CAR-T cell exhaustion, a stepwise loss of effector functions leading to T cell dysfunction [38]. For example, 4-1BB CAR-T cells expressed lower levels of exhaustion markers, such as PD-1, TIM-3, and LAG-3, while producing higher levels of pro-inflammatory cytokines such as IFN-γ, TNF-α, and IL-2 [16]. Regarding their therapeutic effect, G2 PD-L1-targeting 4-1BB-inserted CAR-T cells had superior anti-tumor activity over CD28- and GITR-incorporated CAR-T cells in mouse models of melanoma and lymphoma [18]. Correspondingly, exhaustion-related transcription factors (TBX2a, EOMES, and PRDM1) and inhibitory receptors (LAG-3, HAVCR-2, and CTLA-4) were downregulated upon the global transcriptional profiling of 4-1BB CAR-T cells compared to CD28 CAR-T cells. In addition, several factors expressed in memory T cells (KLF6, JUN, and JUNB) were upregulated [16].

This increase in CAR-T cell persistence has at least been partly attributed to the 4-1BB-dependent induction of non-canonical nuclear factor kappa B (nc NF-κB) signaling [16,17], which is centrally regulated by the NF-κB-inducing kinase (NIK). Specifically, by reflecting RelB nuclear occupancy, a way to assess nc NF-κB signaling, it was found that basal nc NF-κB signaling is 1.3-fold greater in 4-1BB CAR-T cells than in CD28 CAR-T cells. After 12 h of stimulation with target beads coated with anti-CD19 idiotype, several indicators of nc NF-κB signaling increased in 4-1BB CAR-T cells compared to CD28 CAR-T cells, including p100 phosphorylation (3.2-fold), p52/p100 ratio (2.4-fold), and RelB nuclear translocation (5.4-fold) [17]. These characteristics could explain the longer persistence of 4-1BB-inserted CAR-T cells compared to CD28-inserted CAR-T cells. Interestingly, the expression of a constitutively active full-length 4-1BB construct together with a CD28 CAR construct (2028Z-4-1BB CAR-T cells) also increased NF-kB signaling, CAR-T cell viability as well as in vivo persistence and anti-tumor activity [39]. The reduction of nc NF-κB signaling by the overexpression of the C terminus of NIK, which yielded a dominant negative NIK (dn NIK), decreased CAR-T expansion and increased the expression of pro-apoptotic molecules in 4-1BB CAR-T cells [17].

Moreover, it was recently demonstrated that the LCK-mediated constitutive phosphorylation of the CAR CD3ζ domain promotes a higher activation of the CD28 co-stimulated CAR-T cells than 4-1BB co-stimulated CAR-T cells [40]. LCK is recruited to the CAR synapse by either CD8 or CD4 co-receptors. Interestingly, the THEMIS-SHP1 phosphatase complex was recruited to the CAR synapse in CAR-T cells encoding 4-1BB and counteracted the effect of LCK to attenuate CAR CD3ζ phosphorylation. To overcome limited LCK-kinase activity due to the presence of THEMIS-SHP1, CAR-T cells encoding 4-1BB were engineered to co-express LCK. This resulted in better control of tumor growth in NSG mice engrafted with the neuroblastoma tumor cell line CHLA-255 as compared to CD28 co-stimulated CAR-T cells without co-expressing LCK. These effects were observed without an increase in the expression of exhaustion markers or a compromise in persistence.

Collectively, a 4-1BB signaling domain improves the persistence and long-term survival of CAR-T cells compared to other co-stimulatory domains such as CD28. However, 4-1BB-mediated co-stimulation in CAR-T cells may also pose some drawbacks compared to CD28 co-stimulation. For instance, CD28 CAR-T cells were able to overcome regulatory T cell-mediated immunosuppression, whereas 4-1BB CAR-T cells were not [41]. Furthermore, tonic 4-1BB signaling in CAR-T cells can lead to TRAF2-dependent activation of the NF-κB pathway, which may lead to CAR-T cell deletion via Fas-mediated activation-induced cell death [42]. This indicates that different aspects of each co-stimulatory domain should be considered when selecting a strategy to prolong CAR-T cell survival.

As of today, three 4-1BB CAR-T cell products, namely Tisagenlecleucel (Kymriah), Lisocab tagene Maraleucel (BREYANZI), and Idecabtagene Vicleucel (ABECMA), have been approved by the FDA. Tisagenlecleucel, a CD19-targeting G2 CAR-T cell product, was tested in 93 patients with DLBCL where it elicited robust efficiency (52%, ORR; 40%, CR; 12%, PR). Moreover, strong but manageable grade 3 or 4 side effects were observed, such as reversible cytokine release syndrome (CRS) in 89.7% of the patients and reversible neuropsychiatric events in nine patients (31%). In this study, 3 out of 93 patients died from disease progression [2,43]. As there are substantial differences in study designs and patient populations compared to trials with CD28 G2 Yescarta (ZUMA-1, 83%, ORR; 58%, CR) and no formal comparison studies have been performed, no hard conclusions can be drawn from treatment comparisons so far [44].

Lisocabtagene Maraleucel is another CD19-directed G2 CAR-T cell product harboring a 4-1BB domain that additionally expresses a truncated version of the epidermal growth factor receptor (EGFR). The latter can be used to delete CAR-T cells using Cetuximab when required in view of toxicity [3,45,46]. Lisocabtagene Maraleucel treatment in 256 relapsed or refractory large B cell lymphoma patients yielded an ORR of 73% and 53% CR [3]. More importantly, since CD4 and CD8 CAR-T cell subsets are separately produced in Lisocabtagene, a precise and consistent 1:1 CD4:CD8 CAR-T cell ratio is provided in the final product. In contrast, Tisagenlecleucel as well as Yescarta are generated from a bulk T cell population, where inter-patient variation in the CD4:CD8 ratio of the final product may occur. However, this has not yet directly been correlated to response.

A third 4-1BB CAR-T cell product, Idecabtagene Vicleucel, the first FDA-approved CAR-T cell therapy for multiple myeloma, is targeting the B cell maturation antigen (BCMA) expressed on multiple myeloma cells. Idecabtagene Vicleucel yielded responses in 73% of the patients with refractory and relapsed multiple myeloma (rrMM), and a promising 33% achieved a complete response [47]. Additionally, 26% of all patients in this phase 2 study achieved a minimal residual disease (MRD)-negative status. A phase 3 study comparing Idecabtagene Vicleucel vs. standard regimens in disease refractory patients is ongoing (NCT03651128) [48]. Impressively, in triple-class exposed rrMM patients whose preceding therapy consisted of different combinations of immunotherapies, proteasome inhibitors or chemotherapy, and dexamethasone, Idecabtagene significantly improved ORR (76.4% vs. 32.2%), PFS (11.6 vs. 3.5 months), and OS (20.2 vs. 14.7 months) compared to (index) line therapy [49].

In order to combine T cell effector functions with T cell memory induction and to achieve more durable clinical responses, G3 CAR-T constructs comprising both CD28 and 4-1BB co-stimulatory domains have been developed. Compared to G2 CAR-T cells, treatment with G3 CAR-T cells triggered more phosphorylation of important intracellular signaling molecules such as LAT, ZAP-70, SYK, and ERK [50]. Consequently, G3 CAR-T cells produced more IL-2 and had higher proliferative capacity and better in vivo persistence [12,50]. On the other hand, both G2 and G3 CAR-T cells upon antigen stimulation similarly induced CD3/CD8 lineage populations, the expression of exhaustion phenotypes (e.g., PD-1/TIM-3 double-positive), and memory/effector status (based on CD45RA/CCR7 expression) [50]. Similarly, no difference was observed in CD19-directed CAR-T-mediated cytotoxicity between G2 and G3 CAR-T cells in co-culture with CD19-positive DAUDI cells in vitro or in mice. Thus, no major differences between G2 and G3 CAR-T cells appears to exist in cytotoxic activity, although a longer persistence of G3 CAR-T cells may pose significant clinical benefit [50].

Both CD19-targeting CD28 G2 CAR-T cells and G3 CAR-T cells with both CD28 and 4-1BB co-stimulatory domains were tested in patients with relapsed or refractory non-Hodgkin’s lymphoma (NHL) in a dual G2/G3 CAR-T cell product injection study (NCT01853631), with a ~55% ORR reported in the targeted population [51]. In this mixed infusion product, the population of CD4 and CD8 central memory T cells (Tcm) was increased in the G3 CAR-T cell fraction compared to the G2 CAR-T cell fraction at the time of infusion. Interestingly, the G3 CAR-T cells exhibited superior expansion (up to 40-fold) and persistence (at 6 weeks and 6 months) in vivo compared to CD28 G2 CAR-T cells, which was particularly evident in patients with low disease burden [51]. This suggests that combining the 4-1BB co-stimulatory domain in G3 CAR-T cells can synergize with other co-stimulatory domains. Moreover, G3 CAR-T cells may be particularly suited for patients with no measurable disease but with a high risk of relapse after autologous stem cell transplantation (ASCT), as the difference in G2 and G3 CAR-T cell expansion was the largest in patients in remission post-ASCT.

The application of 4-1BB-mediated co-stimulatory signaling in combination with T cell co-stimulatory domains other than CD28 has been investigated as well, such as the combination with an inducible T cell co-stimulator (ICOS) co-stimulatory domain [19]. ICOS is a member of the B7 family and is expressed on activated T cells, facilitates T/B cell co-stimulation, and enhances the production of anti-tumor cytokines (e.g., IFN-γ) [52]. ICOS G2 CAR-T cells produced higher levels of Th1/Th17 signature cytokines, such as IL-17A, IL-17F, and IL-22, than CD28 or 4-1BB G2 CAR-T cells [19]. Combining ICOS with 4-1BB in a G3 4-1BB/ICOS CAR-T cell improved in vivo anti-tumor activity and CAR-T cell persistence in NSG mice bearing pancreatic tumors (Capan-2) as compared to 4-1BB- or ICOS G2 CAR-T cells [19]. Interestingly, this improvement in activity was the strongest when the ICOS domain was proximal to the cell membrane and was combined with its transmembrane domain. A similar configuration with the CD8α transmembrane domain had anti-tumor activity that was comparable to 4-1BB/ICOS CAR-T cells with the 4-1BB domain in the proximal position and a CD8α transmembrane domain. Thus, not only the type but also the location of the co-stimulatory domain as well as the choice of the transmembrane domain in CAR constructs may have an impact on the efficacy of co-stimulatory signaling of a CAR.

Several studies have explored the clinical application of 4-1BB G3 CAR-T cells, with a small trial in four relapsed indolent B cell or mantle cell lymphoma (MCL) patients to test the safety of CD20-targeting CD28 and 4-1BB G3 CAR-T cells that were already reported in 2012 [53]. The inclusion of more co-stimulatory domains was thought to yield more toxicity, but the treatment regimen proved to be generally safe and well-tolerated. Notably, two out of four patients had no detectable disease and remained progression-free for 12 and 24 months, with CAR-T cells being detectable in the blood even 1 year after infusion [53]. G3 CAR-T cells directed at other targets have also been evaluated. For instance, prostate cancer target antigen (PSMA) G3 CD28 and 4-1BB CAR-T cells had potent anti-tumor activity, including the induction of CAR-T cell-mediated tumor killing and ablation of PSMA-positive vessels in tumor sites [54]. Furthermore, patients are currently being recruited for a phase 1 trial to test the safety and efficiency of G3 CD19 CAR-T cells containing 4-1BB and CD28 co-stimulatory domains in adults with relapsed or refractory CD19-positive B acute lymphoblastic leukemia [12].

In conclusion, the inclusion of a 4-1BB co-stimulatory domain in CAR-T cell constructs improved the in vivo persistence of CAR-T cells, enhanced memory formation, and reduced the expression of exhaustion markers. In G3 CAR-T cell constructs combining 4-1BB with CD28 co-stimulation, a higher proliferative capacity and better in vivo persistence was achieved compared to 4-1BB- or CD28 G2 CAR-T cells. Whereas no direct potentiation of cytotoxicity was observed in these G3 CAR-T cells, their longer persistence could improve their clinical benefit, although evidence supporting this statement is still lacking. Notably, the inclusion of an additional co-stimulatory domain could potentially lead to more or stronger treatment-related toxicities, but CD28 and 4-1BB G3 CAR-T cells have so far been found to be safe and well-tolerated. In a recent review, CD28- or 4-1BB-co-stimulated CAR-T cells were reported to be not that different regarding their efficacy in clinical trials, although differences in pre-clinical outcomes were observed (e.g., higher cytokine release from CD28 CAR-T cells) [55]. Comparison of clinical efficacy is challenging due to multiple variations in the clinical studies, including the cohort size of patients, inclusion of different types of cancers, the various sources of co-stimulatory domains, and the choices of the scFv, hinge, and transmembrane domains. Therefore, care should be taken before drawing definite conclusions considering the application of certain co-stimulatory domains in CAR-T cell applications to treat a particular type of cancer. Nevertheless, combining non-overlapping co-stimulatory activity profiles with 4-1BB as a backbone is of clear interest to further augment CAR-T cell therapy.

## 3. OX40 as Co-Stimulatory Domain in CAR-T Cells

Another TNFRSF member being explored as co-stimulator for CAR-T cells is OX40 (for an in-depth review of its biological function, see [56]). In brief, OX40 is upregulated on T cells only upon MHC-TCR engagement. OX40 signaling increases T cell persistence and induces memory T cell (Tm) formation [57,58,59]. OX40 signaling also triggers clonal expansion and upregulates the expression of anti-apoptotic proteins such as Bcl-2, Bcl-xL, and Bfl-1, which promotes the survival of activated T cells [60,61,62]. The latter feature probably proceeds through the activation of the nc NF-κB signaling pathway [63]. In respect of these features, OX40 and 4-1BB share many similarities in their functions, such as an increase in memory T cell response and the limited activation of naïve T cells, but clear difference in activity have been reported in terms of modulating CD4 and CD8 T cell responses [64,65,66,67]. OX40 and 4-1BB actually had contrasting roles in regulating the clonal expansion of CD8 T cells upon adenoviral infection, with OX40-deficient CD8 T cells displaying reduced CD8 T cell expansion, whereas 4-1BB-deficient CD8 T cells actually displayed hyper-responsiveness, resulting in enhanced CD8 T cell numbers. Furthermore, T cell differentiation into functional effector cells required OX40, whereas 4-1BB was responsible for a reduced level of differentiation at an early-time of priming [66]. Thus, although similar, OX40 and 4-1BB also regulate different aspects of T cell activation and differentiation, with OX40 playing an essential role in promoting T cell differentiation and expansion.

When compared to CD28 G2 CAR-T, OX40 G2 CAR-T cells produced more IFN-γ, equal levels of IL-2, and similar CAR-T cell proliferative responses [68]. More importantly, OX40 G2 CAR-T cells produced significantly lower levels of IL-10, a potent anti-inflammatory molecule detected in high levels in the tumor micro-environment that can repress anti-cancer responses (as reviewed in [69]). Correspondingly, a combination of CD28 co-stimulation with OX40 in G3 CAR-T cells directed against carcinoembryonic antigen (CEA) reduced the level of IL-10 compared to CD28 G2 CAR-T cells and prevented the auto-repression of CAR-T cells [68,69,70,71]. Furthermore, CD28/OX40 co-stimulation rescued CAR-T cells from activation-induced cell death upon repetitive antigen engagement [72]. In line with this, CD30-directed CD28/OX40 G3 CAR-T cells had superior cytotoxic activity compared to CD28/4-1BB G3 CAR-T cells in long-term in vitro co-cultures and produced higher levels of Th1 cytokines (e.g., IFN-γ, IL-2, and TNF-α) [73]. In line with these in vitro findings, OX40/CD28 G3 CAR-T cells also displayed longer persistence in vivo compared to 4-1BB/CD28 G3 CAR-T cells [74]. In a similar approach, CLL1-targeted OX40/CD28 G3 CAR-T cells effectively targeted CLL-1-positive AML cells in vitro [75]. These findings were confirmed in a separate study in which a single CD28 co-stimulatory domain was most effective in triggering immediate cytotoxicity, but the inclusion of 4-1BB or OX40 co-stimulation yielded a longer lasting anti-tumor response. In this setting, OX40 co-stimulation proved to be superior to 4-1BB co-stimulation [71].

In a separate approach, 12 full-length and constitutively expressed co-stimulatory receptors (including 4-1BB, CD40L, ICOS, GITR, HVEM, CD27) were evaluated for their stimulatory activity on 4-1BB G2 CAR-T cells. The activation of these receptors was dependent on engagement by their endogenous co-stimulatory ligand rather than antigen recognition by the CAR. The OX40-expressing 4-1BB G2 CAR-T construct more robustly enhanced CAR-T cell proliferation and anti-cancer cytotoxicity than any of the other CAR-T constructs preclinically [20]. The OX40 CAR-T construct also reduced the apoptotic AICD-mediated elimination of T cells and prevented T cell exhaustion. In another recent report, several co-stimulatory combinations were screened for their cytolytic activity within a G2 tri-specific CD19-CD20-CD22-targeting CAR-T cell approach, with OX40, ICOS, or CD27 co-stimulatory domains having the most potent response upon antigen recognition [10]. These domains outperformed the co-stimulatory domains CD28 or 4-1BB.

Taken together, although still lagging behind 4-1BB, early evidence suggests that OX40 co-stimulation is a promising co-stimulatory domain for use in CAR-T cells. Its prevention of auto-repression, rescue from activation-induced cell death, and its superior cytotoxic activity are encouraging features. In this respect, a CD28/OX40 CAR-T cell targeting disialoganglioside (GD2)-positive solid cancers, such as melanoma, neuroblastoma, osteosarcomas, and some other sarcomas, is the first OX40-based G3 CAR-T cell being evaluated in a clinical trial for the treatment of metastatic melanoma (NCT02107963).

## 4. CD27 as Co-Stimulatory Domain in CAR-T Cells

The expression of CD27 is found on CD4 and CD8 T cells, NK cells, and primed B cells and facilitates co-stimulatory signaling to these cells [76]. CD70, or CD27 ligand, is expressed on antigen-presenting cells, including DCs and macrophages. Upon ligation of CD27 on T cells, downstream signaling is induced through NF-κB-inducing kinase, resulting in activation, differentiation, and clonal expansion. In this respect, TNFRSF member CD27 has also recently been explored as co-stimulatory domain in CAR-T cells. CD27/CD70 interaction normally boosts T cell activation, differentiation, and promotes the clonal expansion of effector T cells, features for which CD27 targeting is being exploited in antibody-mediated anti-tumor strategies [77,78]. CD27 G2 CAR-T cells had enhanced antigen-stimulated effector functions, upregulated expression of anti-apoptotic proteins (e.g., Bcl-X(L)), increased in vivo persistence, and enhanced tumor regression in an SKOV3 human ovarian cancer cell mouse model compared to G1 CAR T cells [79]. In this model, no differences in tumor regression were observed between CD27 G2 CAR-T cells and 4-1BB or CD28 G2 CAR-T cells. Nevertheless, CD27 co-stimulation, similar to 4-1BB co-stimulation, augmented CAR-T cell persistence compared to CD28-mediated co-stimulation [79]. These findings were recently confirmed for NKG2D-targeting CAR-T cells in a triple-negative breast cancer model [80].

In a recent study, a library of CAR-T cell constructs with several co-stimulatory domains was evaluated, with the combination of CD27 and DAP-10 yielding the best anti-tumor response in vitro against 24JK erbB2-positive sarcoma cells as well as in vivo in NOD-SCID mice [81]. DAP-10 promotes T cell activation and T cell-mediated cytotoxicity and has therefore been implemented in other CAR-T cell-based studies as well [82,83]. Interestingly, even though eliciting the strongest anti-tumor response, the DAP-10/CD27 G3 CAR-T cells tended to be present at lower frequencies compared to CD28 G2 CAR-T cells in vivo. The reason is yet to be elucidated but might be a reflection of the lower survival of T cells expressing a more cytotoxic CAR construct.

Clinically, treatment with a GD2-targeting CD27/CD28 CAR-T cells yielded 15% PR and 38% SD in 34 neuroblastoma patients, with a one-year survival rate of 74% (NCT02992210 and NCT02765243) [84]. In another recent clinical study with CD27/CD28 CAR-T cells, CLL1-targeted CAR-T cells proved to be safe in AML patients [85]. Impressively, in this study, three out of four patients had a CR and were MRD-negative and eligible to proceed to hematopoietic stem cell transplantation (HSCT). Side effects in these patients were limited to grade 1 to 2 CRS. In a multi-CAR-T cell therapy regimen study designed to prevent antigen-negative relapses, primary, booster, and/or consolidation CAR-T infusions targeting CD19, CD22, CD30, GD2, and/or PSMA (all with co-stimulatory signals for CD28 and CD27) were developed based on the evaluation of pre-treatment and during treatment antigen expression of tumor biopsies of lymphoma patients [86]. Notably, four patients with relapsed/refractory B cell lymphomas, all receiving a patient-tailored CAR-T cell combination, achieved prolonged remissions (>1 year) with very low treatment-related toxicities. This innovative combinational CAR-T cell approach requires follow-up studies to determine its full potential and long-term effects, but is a prime example of personalized and treatment-tailored CAR-T cell therapy.

Worthy of note is the fact that CD70 is also aberrantly expressed on a number of hematological malignancies, leading to the design of CD70-targeting CAR-T cells with a full-length endogenous protein ligand CD27 as the antigen-recognition domain combined with a CD3-ζ chain [87]. These CD70-specific CAR-T cells not only interact with CD70-expressing hematological malignant cells, but can also be activated via CD27-mediated co-stimulatory signaling upon antigen recognition, depending on a TRAF2/5-binding site within the cytoplasmic CAR tail. These CAR-T cells recognize and eliminate primary B and T cell lymphomas in vitro and induce the regression of established CD70-positive lymphoma in mice. In preclinical Molm-13 and THP-1 AML xenograft mouse models, CD70-inserted CAR-T cells were significantly more effective than CD70scFv G2 CAR constructs (either with CD28, 4-1BB, or CD27 domains) [88]. CD70 CAR-T cells also exhibited higher levels of T cell proliferation in vivo compared to CD28 CAR-T cells. Furthermore, compared to 4-1BB, CD28 or CD27 co-stimulated CD70scFv CAR constructs, TNF-α and IFN-γ secretion was increased in CD27-ligand CAR-T cells, and the expression of exhaustion markers LAG3, TIM-3, and PD-1 was the lowest in in vitro assays.

As a note of caution, the ligation of CD27 can also induce terminal differentiation, exhaustion, and apoptosis of CD8 T cells [89]. Indeed, a constitutively expressed CD27 molecule on the surface of IL13 receptor α2-targeting G2 4-1BB CAR-T cells greatly reduced the proliferation and induced the exhaustion and apoptosis of CAR-T cells [90]. Nevertheless, sorting of the endogenous CD27-positive CAR-T cell population yielded a CAR-T preparation that outperformed CD27-negative CAR-T cells in orthotopic glioblastoma xenografts. The CD27-positive CAR-T cells displayed a memory-associated gene signature and were characterized by lower exhaustion levels compared to CD27-negative counterparts [90]. Upon the targeting of CD70-expressing tumor cells, CD27 expression on CAR-T cells was quickly downregulated, suggesting that chronic CD27 stimulation is potentially deleterious.

In conclusion, CD27-mediated co-stimulation can enhance different CAR-T cell features such as CAR-T cell persistence and cytotoxicity. Clinically, the CD27/CD28 co-stimulatory combination yielded impressive responses in neuroblastoma, AML, and lymphoma patients. In addition, exploiting the endogenous protein ligand CD27 as the antigen-recognition domain in CAR-T cells is a very promising development for CD70-positive cancers. However, a combination of CD27 and other co-stimulatory molecules in CAR-T cell construct should also be considered carefully to avoid terminal differentiation and exhaustion, and CD27 should therefore not be constitutively activated.

## 5. CD40 as Co-Stimulatory Domain in CAR-T Cells

TNFRSF member CD40 is expressed on myeloid-derived antigen-presenting cells such as dendritic cells (DCs), granulocytes, and macrophages [91,92]. The CD40 ligand (CD40L) is mainly expressed on activated T cells and NK cells [93,94]. On APCs, the ligation of CD40 by CD40L licenses antigen presentation and co-stimulatory signaling, which subsequently promotes the activation and expansion of cytotoxic T cells [93,95,96]. Although CD40 agonism is well-described as a stimulator of antigen-presenting cells, its direct effect on T cells is less well-characterized. So far, signaling via CD40 expressed on T cells has been reported to augment CD3/CD28 agonism via NFAT signaling and promote IL-2 secretion [97]. Moreover, CD40 signaling in activated CD8 T cells regulated T cell memory and ameliorated CD8 CTL exhaustion, as discussed in detail in [98]. CD40-mediated signaling is transduced via various TNF receptor-associated factors (TRAFs), with five out of seven recognized member proteins in the TRAF family (TRAF 1–3,5, and 6) reported to form TRAF homotrimers or hetero-oligomers to interact with the cytoplasmic domain of CD40 [99]. TRAFs are recruited to the CD40 trimer and subsequently trigger the downstream signaling of CD40 [100]. In particular, TRAF2/3 are reported to be essential for CD40-mediated downstream signaling, with CD40 ligation resulting in their translocation into membrane lipid rafts [101]. Triggering of CD40-mediated co-stimulation in CAR-T cells upon antigen recognition could therefore have beneficial immuno-stimulatory effects. Moreover, artificial overexpression of CD40L on the surface of CAR-T cells can also stimulate the endogenous immune system via the CD40L-mediated activation of APCs and the subsequent priming of endogenous T cell responses [102]. Thus, the CD40/CD40L axis can play an interesting dual role in CAR-T cell approaches.

In initial studies, no differences were observed in antigen-specific target cell killing between CD40 or 4-1BB CAR-T cells in neither the G2 format nor the G3 format with a CD28 domain [103]. However, CD40 co-stimulation did trigger stronger NF-κB activation compared to 4-1BB co-stimulation [103]. To expand on these findings and possibly augment co-stimulatory signaling, CD40 CAR-T cells have been combined with other co-stimulatory molecules in CAR constructs, for example, with B cell signaling moiety CD79A. Both receptors activate downstream NF-κB, NFAT, and AP-1 signaling [104], for which signal integration and synergistic activation are expected upon combination of these domains in CAR-T cells. Indeed, in in vitro co-cultures with CD19-expressing cells, CD79A/CD40 CAR-T cells had elevated NF-κB and p38 activity and proliferated more compared to CD28 or 4-1BB co-stimulated CAR-T cells. CD79A/CD40 co-stimulated CAR-T cells also had superior anti-tumor activity and proliferative activity compared to CD28 or 4-1BB co-stimulated CAR-T cells in mice inoculated with Raji tumor cells [105]. Moreover, in the aforementioned combinatorial library screen of 700k intracellular domains to check for optimal ICD composition in CD19-targeted G3 CAR-T cells, the combination of CD40, CD3ε ITAM, and DAP-12 recently emerged as the most efficient. This was based on more persistent proliferative activity, enhanced tumor control, and reduced exhaustion compared to 4-1BB G2 CAR-T cells in vitro [11]. However, no significant improvement of this combination over 4-1BB G2 CAR-T cells was observed in xenograft mouse models.

CD40 co-stimulation was further combined with a myeloid differentiation primary response 88 (MYD88) domain, although in this case not incorporated into the CAR construct but as a separate signaling moiety [106,107]. Physiologically, MYD88 is an important adaptor molecule in Toll-like receptor (TLR) signaling that facilitates CD40-mediated signaling in myeloid cells [108]. To therapeutically exploit this signaling, an inducible MyD88/CD40 fusion protein (termed iMC) was constructed, which comprised a membrane-targeting myristoylated peptide, truncated MyD88, and the cytoplasmic domain of CD40. Co-stimulatory signaling by this fusion protein requires cross-linking, which can be experimentally triggered by the homodimerizing compound Rimiducid, normally used to activate the inducible caspase 9 (iC9) suicide gene in CAR-T cells [109]. Compared to CAR-T cells without an incorporated iMC domain, iMC-based CAR-T cells had better in vivo engraftment, proliferation, and stronger anti-tumor effects in various xenograft mouse models, including lung cancer, metastatic osteosarcoma, AML, and pancreatic adenocarcinoma [106,107]. In a phase 1/2 clinical trial, treatment with prostate stem cell antigen (PSCA)-targeting and iMC-containing CAR-T cells (named BPX-601) yielded a stable disease (SD) in three out of five advanced pancreatic cancer patients (NCT02744287) [110]. BPX-601 cells persisted, expanded in vivo, enhanced serum levels of key cytokines such as IFN-γ and GM-CSF, and infiltrated in metastatic lesions in patients.

The iMC concept has also been applied to CAR-modified NK cells, coupled with ectopically expressed interleukin-15, yielding enhanced anti-tumor activity of CD123-specific or BCMA-specific CAR-NK cells in NSG mice engrafted with THP-1 cells as compared to non-iMC-based CAR-NK cells [111]. Alternatively, a CD19-targeted and CD123-targeted CAR-T cell platform was developed with “always on” MyD88/CD40 signaling, which yielded robust anti-tumor activity in lymphoma and leukemia mouse models. To avert toxicity by these highly active CAR-T cells, a Rimiducid iC9 suicide gene safety switch was introduced to allow for CAR-T ablation. Consequently, excessive activity/toxicity was quickly and effectively resolved in mouse models [112].

Interestingly, by artificially expressing the CD40L as constitutive co-stimulatory molecule on the surface of CAR-T cells, the priming and clonal expansion of endogenous tumor-reactive T cells can be facilitated via the stimulation of CD40-expressing APCs [102]. In an immunocompetent lymphoma mouse model, CD19-targeting G2 CD40L-expressing CAR-T cells produced significantly more effector cytokines (e.g., TNF-α, IFN-γ). Thus, the endogenous tumor-reactive T cell effector function was increased, with more IFN-γ/TNF-α-positive CAR-negative T cells present in both the tumor and the spleen as compared to mice treated with CAR-T cells without CD40L [102]. Upon post-treatment sorting of endogenous non-CAR T cells from mice treated with CD40L expressing CAR-T cells, in vitro re-stimulation triggered enhanced IFN-γ secretion as compared to CAR-T cell treatment without CD40L. In particular, CD40L-expressing CD19 G2 CAR-T cells displayed potent anti-tumor activity in CD19/CD40 double-positive A20 lymphoma-bearing BALB/c mice as well as in mice with CD40-negative leukemia cells, proving that the CD19-targeted CD40L-expressing CAR-T cells also function without CD40/CD40L-mediated cytotoxicity [102]. Indeed, CD40L-induced signaling appears to help increase effector cytokine production and reverse a suppressive tumor microenvironment.

Taken together, although its direct effect on T cells have been less established, the superiority of CD40 co-stimulation over 4-1BB has been revealed in G2 CAR-T cells. Furthermore, the promising results found in combination with signal moiety CD79A and MyD88 demonstrate that CD40/CD40L signaling holds clear promise for the modulation of CAR-T therapy. The artificial expression of CD40L on the surface of any tumor-targeted CAR-T cell further provides an additional interesting opportunity to exploit CD40/CD40L signaling to stimulate endogenous anti-cancer T cell immunity.

## 6. HVEM and GITR as Co-Stimulatory Domains in CAR-T Cells

Two additional members of the TNFRSF—HVEM and GITR—have also recently been incorporated into CAR-T cell constructs. HVEM has both immunostimulatory and immune inhibitory functions and interacts with the ligands CD160, BTLA (B and T lymphocyte attenuator), LIGHT, and lymphotoxin α (LTα) [113,114,115]. HVEM is expressed on various immune cells, including T cells, B cells, NK cells, and dendritic cells. The binding of HVEM to LIGHT or LTα leads to T cell activation and proliferation [113,115,116], whereas the binding of HVEM to T cell-expressed BTLA inhibits T cell activation. Furthermore, HVEM also regulates effector and memory formation in CD8 T cells [117,118].

So far, HVEM has only been reported once as a co-stimulatory domain in CAR-T cells, with HVEM G2 CAR-T cells exhibiting enhanced anti-tumor cytotoxicity and pro-inflammatory cytokine production (e.g., IL-2, TNF-α, and IFN-γ)) as compared to control CD28 or 4-1BB G2 CAR-T cells [119]. Moreover, CAR-T exhaustion was reduced in HVEM G2 CAR-T cells, and equivalent percentages of Tcm and Tem cells were detected. In contrast, in CD28 and 4-1BB CAR-T cells the Tem and Tcm subsets, respectively, were highly enriched. Thus, HVEM G2 CAR-T cells appear to yield a more balanced CAR-T cell phenotype. Interestingly, enhanced levels of glycolysis and mitochondrial respiration were detected in HVEM CAR-T cells in vitro [119]. Thus, HVEM co-stimulation appears to increase energy metabolism, a feature positively related to reduced T cell exhaustion [120,121].

Another TNFRSF co-stimulatory member beginning to be explored for CAR-T co-stimulation is GITR. Unlike 4-1BB or OX40, GITR is constitutively expressed on T cells, with a higher expression on CD4/CD25/Foxp3-positive Tregs as compared to naïve or memory T cells [122]. Furthermore, the expression of GITR is upregulated on effector T cells upon TCR activation [123]. GITR agonists inhibit the suppressive activity of Tregs and improve the survival of effector T cells [124,125,126]. In CAR-T cells, GITR co-stimulation yielded a comparable or even more robust anti-tumor activity than CD28 or 4-1BB G2 EGFR-targeted CAR-T cells in various tumor cell lines, including SKOV-3, A1847, and BxPC3 [127]. In addition, CD19-targeting GITR G2 CAR-T cells also significantly suppressed Raji tumor cell growth in vivo, although no comparison to other CAR constructs was performed [127]. Moreover, compared to the co-stimulatory CAR-T domains DAP-10, CD28, 4-1BB, ICOS, and OX40, GITR co-stimulation exhibited stronger cytotoxicity in vivo in T cell lymphoma and melanoma models [18]. However, compared to CD28 CAR-T cells, GITR co-stimulation did reduce the secretion of TNF-α, IL-2, and Th17-associated cytokines and induced a more differentiated effector phenotype combined with reduced in vivo persistence [18]. Taken together, this could indicate that GITR co-stimulation facilitates the dominance of T effector cells, which suggests that a combination with a 4-1BB co-stimulatory domain to enhance in vivo persistence, enhance memory formation, and reduce the expression of exhaustion markers may be a worthwhile combination to be evaluated as a novel G3 CAR-T cell approach.

## 7. Conclusions

**Figure 2:**
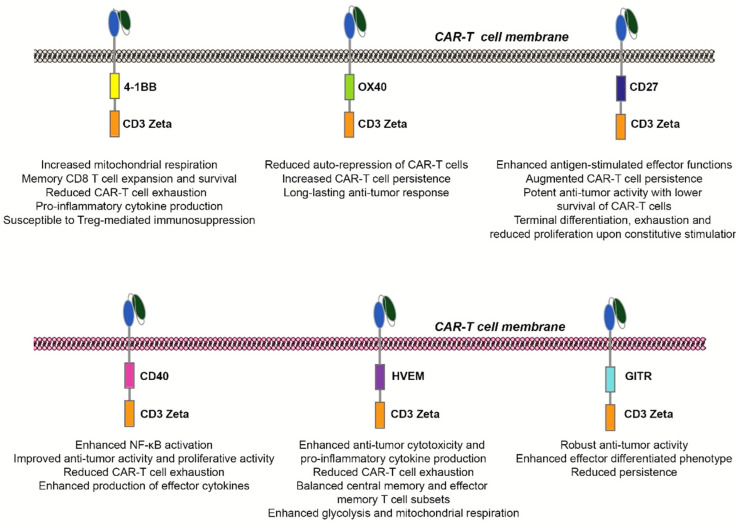
Unique functional roles of selected co-stimulatory members of the TNFRSF in CAR-T cell therapy. Both overlapping and distinctive features of 4-1BB, OX40, CD27, CD40, HVEM, and GITR in CAR-T cell constructs are highlighted.

In this review, we have detailed the various co-stimulatory members of the TNFRSF being implemented in the tailoring of CAR-T cell activity, specifically highlighting distinct advantages and disadvantages of various TNFRSF members (Figure 2). It is evident that by selecting and/or combining different co-stimulatory domains, one can greatly influence and drive CAR-T cell behavior. In the ongoing quest for the selection of co-stimulatory domains in CAR-T cells, a large-scale library-based screening of intracellular domains (comprising ~700k options) in G3 CAR-T may help delineate an optimal combination of co-stimulatory domains for individual targets/diseases and constructs.

In this respect, the CD28 and 4-1BB co-stimulatory domains typically used in clinical products were evidently outperformed by multiple co-stimulatory domains, including OX40, ICOS, or CD27, in a recent side-by-side comparison [10]. Thus, further engineering of the intracellular domain of CAR-T cells is likely to yield enhanced functionalities. For instance, the OX40 co-stimulatory domain, similar to 4-1BB, can be used to enhance CAR-T cell persistence but provides a reduced activation of effector functions compared to the CD28 co-stimulatory domain. The GITR co-stimulatory domain may be used to drive effector functions but, as CD28, is associated with reduced persistence. Furthermore, in terms of eliciting a long-lasting anti-cancer response, OX40 co-stimulation proved to be superior to 4-1BB co-stimulation. Even though its direct effect on T cells has not yet been completely elucidated, CD40 CAR-T cells also appear to have strong stimulating effects on endogenous immunity. In particular, the combination of CD40 with the TLR adaptor MyD88 within the iMC domain as a second immunomodulatory receptor is an interesting way to augment CAR-T cell activity.

Particularly, the combination of different co-stimulatory domains in G3 or G4 CAR-T cells is likely to yield additional clinical benefits, as different CAR-T cell functions can be combined to yield synergistic activity. For instance, by combining CD28 and OX40 co-stimulatory domains, an earlier effector phenotype is merged with persistence and a long-lasting anti-tumor response, superior to that of CD28/4-1BB-incorporated CAR-T cells [73,74]. In another G3 CAR-T cell approach, CD28 co-stimulation was evaluated in combination with 4-1BB and CD40 domains, with the latter displaying the most potent co-stimulatory effects. Interestingly, the modification of endogenous co-stimulatory domains might also be a strategy to optimize CAR-T cell functioning, as the introduction of a single amino acid point mutation in the CD28 domain augmented in vivo persistence and prevented exhaustion for CD28 CAR-T cells [128]. Similar domain engineering may be incorporated in the workflow of domain optimization for CAR-T cell therapy for TNFR-based CARs, with the consensus binding sites for TRAF binding in the cytoplasmic tail of 4-1BB being known [25,65,129,130] and spaced such that the binding of a TRAF to one site could sterically hinder binding to the other. Here, domain engineering might be applied to augment 4-1BB signaling intensity.

Similarly, as metabolic responses upon signaling via TNFRSF are distinct and therefore yield different oxygen requirements, the inclusion of co-stimulatory domains should be considered carefully when designing CAR-T cells targeting tumor-specific TMEs with high or low oxygen availabilities [15,131,132]. Metabolic screening may also be included to better predict CAR-T cell treatment outcome in different environments. Concerning the TME, the impact of this immunosuppressive environment clearly affects the clinical efficacy of CAR-T cell therapy. It was recently shown that pre-conditioning with cyclophosphamide could favorably modify the immunosuppressive TME, yielding durable curative responses of prostate stem cell antigen-targeting 4-1BB-co-stimulated CAR-T cells in metastatic prostate and pancreas cancer models [133]. As described above, tuning CAR-T may also help modify the TME, with OX40 G2 CAR-T cells producing significantly lower levels of the anti-inflammatory molecule IL-10 than CD28 G2 CAR-T cells. A combination of CD28 co-stimulation with OX40 in G3 CAR-T cells also reduced the level of IL-10 compared to CD28 G2 CAR-T cells and prevented the auto-repression of CAR-T cells [68,69,70,71]. Therefore, a combination of CD28 and OX40 co-stimulation might apply to CAR-T cells targeting an immunosuppressive TME.

In addition to the identification of which co-stimulatory member of the TNFRSF family to include in CAR-T cell constructs, attention should also be paid to the order of the ICD domains as well as which transmembrane domain to include and the design of the hinge/spacer region (as reviewed in [134]). As described above, the positioning of the co-stimulatory domain within the CAR constructs (e.g., ICOS/4-1BB [19]) can greatly influence CAR-T cell functioning and dictate whether added benefit is derived from a co-stimulatory domain. Collectively, several features should be taken into consideration when designing CAR-T cell constructs, especially with the introduction of CAR-T cell constructs containing more co-stimulatory domains such as G3, G4 and possibly G5 constructs.

As the diverse roles of the different TNFRSF members are still being explored, we are left with gaps in our understanding regarding this unique family, and some TNFRSF members have so far been less explored as co-stimulatory domains in CAR-T cell products such as HVEM and GITR. Nevertheless, it is only a matter of time before the full potential of these members is unlocked, alone as well as in combination with other co-stimulatory TNFRSF members. Taken together, it is clear that in the next-generation of CAR-T cells, TNFRSF members will take a central, diverse, and essential role in CAR-T cell functionality.
